# Alterations of core structural network connectome associated with suicidal ideation in major depressive disorder patients

**DOI:** 10.1038/s41398-021-01353-3

**Published:** 2021-04-24

**Authors:** Xinyi Liu, Cancan He, Dandan Fan, Feifei Zang, Yao Zhu, Haisan Zhang, Zhijun Zhang, Hongxing Zhang, Chunming Xie

**Affiliations:** 1 Department of Neurology, Affiliated ZhongDa Hospital, School of Medicine, Southeast University, Nanjing, Jiangsu China; 2 Xinxiang Key Laboratory of Multimodal Brain Imaging, Henan Provincial Mental Hospital, Xinxiang Medical University, Xinxiang, Henan China; 3 Department of Psychiatry, Henan Provincial Mental Hospital, Xinxiang Medical University, Xinxiang, Henan China; 4 Neuropsychiatric Institute, Affiliated ZhongDa Hospital, Southeast University, Nanjing, Jiangsu China; 5 Psychology School of Xinxiang Medical University, Xinxiang, Henan China

**Keywords:** Diagnostic markers, Depression

## Abstract

Suicide ideation (SI) is a most high-risk clinical sign for major depressive disorder (MDD). However, whether the rich-club network organization as a core structural network is associated with SI and how the related neural circuits are distributed in MDD patients remain unknown. Total 177 participants including 69 MDD patients with SI (MDDSI), 58 MDD without SI (MDDNSI) and 50 cognitively normal (CN) subjects were recruited and completed neuropsychological tests and diffusion-tensor imaging scan. The rich-club organization was identified and the global and regional topological properties of structural networks, together with the brain connectivity of specific neural circuit architectures, were analyzed. Further, the support vector machine (SVM) learning was applied in classifying MDDSI or MDDNSI from CN subjects. MDDSI and MDDNSI patients both exhibited disrupted rich-club organizations. However, MDDSI patients showed that the differential network was concentrated on the non-core low-level network and significantly destroyed betweeness centrality was primarily located in the regional non-hub regions relative to MDDNSI patients. The differential structural network connections involved the superior longitudinal fasciculus and the corpus callosum were incorporated in the cognitive control circuit and default mode network. Finally, the feeder serves as a potentially powerful indicator for distinguishing MDDSI patients from MDDNSI or CN subjects. The altered rich-club organization provides new clues to understand the underlying pathogenesis of MDD patients, and the feeder was useful as a diagnostic neuroimaging biomarker for differentiating MDD patients with or without SI.

## Introduction

Major depressive disorder (MDD) is a highly prevalent and potentially debilitating mood disorder that causes severe morbidity and mortality worldwide^1^, and is associated with approximately 31% of life-long suicide attempts^2^. Suicidal ideation (SI) is the main prerequisite for suicidal attempt, and it may take several minutes to several months to commit suicide^3^. A recent study suggested that the lifetime prevalence of MDD patients related to SI is estimated to be 53.1%^4^. Therefore, SI is regarded as a red alert and maybe the primary predictor for suicide in MDD patients^5^. Although several sensitive brain regions in MDD patients in relation to SI have been identified across several decades of research^6,7^, knowledge about whole brain structural network basis associated with SI is still limited. Hence, it is necessary to confirm the existence of structural network characteristics in MDD patients with or without SI.

Previous neuroimaging studies on brain connectome have reported alterations in the connectivity of the characteristic brain networks in MDD patients with SI (MDDSI). These mainly implicated in the reduced functional connectivity of the frontal-thalamic circuit^8^, together with the decreased structural connectivity of the rostral middle frontal cortex, pallidum, superior parietal lobule, frontal pole, caudate nucleus, putamen, and thalamus in the left hemisphere^9^. In the above-mentioned studies, the entire brain is modeled as a network of nodes corresponding to brain regions and a network of edges representing the relationship between any pairs of brain regions^10,11^. In this huge and complex brain network, there is a core network composed of the highly interconnected hub nodes referred to as the “rich-club organization”^12^. Notably, the rich-club organization integrates functional control and information flow, which plays a vital role in the “high-order” topology of brain networks and hierarchical sub-networks^13^. When the rich-club organization is utilized to define the hierarchical sub-networks^14^, more useful transmitted information can be obtained. Our previous study has proved that the rich-club organization was destroyed and relatively sensitive to distinguish MDD patients from healthy control subjects^15^. However, to date, little is known about the alterations of the rich-club organization networks in MDD patients with or without SI. Thus, the current study aims to explore the significance of rich-club organization networks in MDD patients with or without SI by integrating graph-theoretic measurements with the diffusion tensor imaging (DTI) technology.

In addition, the connectivity within- and between depression-related circuits is another research interest in brain connectome. Previous studies have identified that the sub-frontal circuit (including regions related to executive function and impulsivity) involved in emotional processing seems to exert an important part in the generation of SI in MDD patients^9,16^. However, the circuit positioning does not systematically describe the neural circuit architecture in the framework of emotional disorders. For instance, Leanne M Williams detailed six neural circuits related to depression using large-scale connectivity analysis^17^. Notably, these neural circuits may become the biological basis of emotional dysfunction and clinical biotypes to explain the natural heterogeneity of depression. As a result, it is essential to examine the differences in white matter (WM) fibers constructing the rich-club organization and further identify the location of these abnormal neural circuits in MDD patients with and without SI.

As such, we centered on our hypotheses that the reduced topological characteristics of the rich-club organization as a core structural connectome in MDD patients with SI were related to the cognitive or emotional modules, and provided good power for distinguishing MDDSI from MDD without SI (MDDNSI) or cognitively normal (CN) subjects.

## Material and methods

The study was approved by the Ethics Committee of the Second Affiliated Hospital of Xinxiang Medical University. Each participant provided the written informed consent before the study.

### Participants

A total of 129 MDD inpatients and 60 CN subjects (through advertisements) were recruited from the Second Affiliated Hospital of Xinxiang Medical University. Inclusion and exclusion criteria in MDD patients and CNs are described in the Supplementary Materials. Together 129 MDD patients were divided into two groups, including 69 MDDSI and 60 MDDNSI. Here, SI was defined as the thought of engaging in an act designed to end life. A history of SI was confirmed through clinical interview and based on their medical records of suicidal thoughts. In addition, the severity of suicidal ideation is classified (see Supplementary Materials).

All participants in this study were Chinese and right-handed. After excluding participants with over-motion and/or incomplete echo planar imaging (EPI) scans, 69 MDDSI, 58 MDDNSI, and 50 CN subjects were retained in our final analyses.

### Behavior measurements

On the day of DTI scan, the severity of depressive symptoms in all MDD patients and CN subjects was measured using the 17-item Hamilton Depression Rating Scale (HAMD)^18^.

### MRI acquisition and processing

The detailed parameters of acquisition and image preprocessing information are described in the Supplementary Materials.

### Brain network construction

#### Cortical parcellation for network node definition

Individual T1-weighted images were co-registered to b0 images in DTI native space by the affine transformation. Thereafter, the converted T1 images were normalized to the T1 template in MNI space by non-linear transformation. Eventually, the automatic anatomical landmark (AAL) mapping^19^ was applied in distorting MNI space into DTI native space through the inverse transformation.

#### Tractography for network edge definition

Deterministic tractography-beam photography was performed according to the Fiber Assignment by Continuous Tracking (FACT) method, so as to reconstruct the white matter fiber pathways to define the connected edges. The procedure was initiated at the seeds of WM and ended with FA < 0.15 or a voxel with a turning angle > 45°. To reduce the effect of pseudo connection caused by the noise effect, the streamline number > 3 between nodes was defined as the structural connectivity (SC) matrix.

### Network properties

In this study, various graph theory properties were calculated under different thresholds, including normalized shortest path length (L), normalized clustering coefficient (C), and small world (SW), global efficiency (E_g_), local efficiency (E_loc_), rich-club and modularity^10^. Differences between groups of 1,000 matching random networks were distinguished using the sparse threshold, which was defined as the ratio of the actual number of edges to the maximum possible number of edges in the network (range, 0.05–0.20, in the increments of 0.01), Later, these values were integrated to obtain the differences in number between groups. Noteworthy, the minimum threshold should be greater than log (N) (where N is the total number of nodes; *N* = 90 in this study), while the sigma value of all individual networks under the maximum threshold should be greater than 1.1 to guarantee the compliance with SW^20^.

**Fig. 1:**
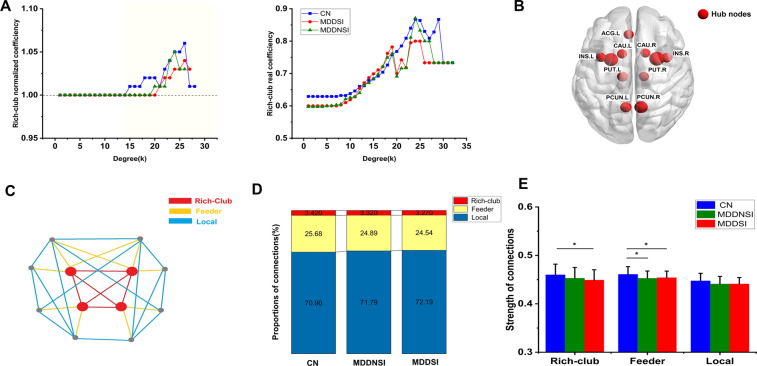
Rich-club organization of structural network. **A** Normalized and real rich club organization curves for the structural group-averaged brain network in CN (blue line), MDDSI (red line), and MDDNSI (olive line) (K > 14, *P* = 0.026, 5000 permutations). **B** Distributions of hub nodes (red nodes) in all individuals. **C** Description model for rich club organization. Structural connections cross individual brain network was divided into 3 distinct subtypes: rich-club (red lines) connections link rich-club members, feeder connections (yellow lines) link rich-club members to non-rich-club members, and local connections (blue lines) connect non-rich-club members. **D** The proportions of rich-club, feeder, and local connections in the structural group-averaged brain networks of each group. **E** The integrated connection strength of each sub-properties (rich-club, feeder, local) of rich club organization. Color icon as before. The error bars indicate standard deviation. ^*^P < 0.05. CN, cognitively normal; MDDSI, major depression disorder with suicidal ideation; MDDNSI, major depression disorder with non-suicidal ideation; INS.L, left insula; INS.R, right insula; CAU.L, left caudate; CAU.R, right caudate; PUT.L, left putamen; PUT.R, left putamen; THA.L, left thalamus; THA.R, right thalamus; PCUN.L, left precuneus; PCUN.R, right precuneus; ACG.L, left anterior cingulate and paracingulate gyrus; FA, fractional anisotropy.

Hub nodes in the rich-club organization are defined according to the nodal properties (including degree centrality), and are screened based on the group-averaged normalized rich-club coefficient calculated through selecting at least 80% of all connections in the subject groups^12^. Notably, the normalized rich-club coefficient Φ_norm(k)_ (Φ_norm(K)_ = Φ(k)/Φ_random_(k)) > 1 indicates the existence of rich-club organization through range of degree^21^. It can be classified into 3 classes, namely, rich club, feeder, and local connections^12^ (Fig. 1). Among them, rich-club connections link the rich-club hubs, and feeder connections link the rich-club hubs with the non-rich-club hubs, whereas local connections link the non-rich-club hubs. The integrated values and proportions of these classes were calculated in this study. Within the range of hub nodes, the regional nodal characteristics, including degree centrality (Dc), nodal efficiency (Ne), and betweenness centrality (Bc), were added into our study. In addition, the asymmetry of hemisphere was explored, and the nodes inside the hemisphere were further divided into two connected subnets corresponding to the left or right hemisphere. Moreover, the metrics were calculated for the left and right hemispheres in each brain region. Then, the number of nodes in the left hemisphere was counted as X (L), whereas that in the right hemisphere as X (R). The measured hemispherical asymmetry was obtained via the following formula: [X (L) − X (R)]/[X (L) + X (R)]. Typically, a positive value in the asymmetry of hemisphere indicates that the asymmetry of the left hemisphere, while a negative value suggests the asymmetry of the right hemisphere^22^.

The GRETNA software (http://www.nitrc.org/projects/gretna/) was employed for all the above network graph theory analyses. Tab. S1 summarizes the definitions of all network indicators.

### Modular analysis of the discontinuous subnetwork edges

In line with the network model of Leanne M Williams^17^, to test those hypotheses associated with the default mode network (DMN), salience circuit (SC), negative affect circuit (NAC), positive affect circuit (PAC), attention circuit (AC), and cognitive control circuit (CCC), masks were created for these six networks based on the AAL atlas (Tab. S2). Of them, the DMN was defined by combining the anterior medial prefrontal cortex, posterior cingulate cortex, with the angular gyrus^23^. Besides, SC was defined by the core nodes in the anterior cingulate cortex (ACC), anterior insula, and sublenticular extended amygdala^24^. The NAC engaged by the negatively valence stimuli was comprised of subcortical nodes in the amygdala, hippocampus, insula, together with both dorsal and ventral prefrontal nodes^25^. At the same time, the PAC engaged by reward-processing was constituted by the striatal nucleus accumbens ventral tegmental areas, along with their projections to the orbitofrontal cortex and the medial prefrontal cortex^26^. Meanwhile, AC was combined by the nodes in the medial superior frontal cortices, anterior insula, anterior inferior parietal lobule, and precuneus^27^. The CCC was made up of nodes in the dorsolateral prefrontal cortex, ACC, dorsal parietal cortex, and precentral gyrus^28^. The full names of these regions are listed in Supplementary Tab. S2.

### Statistical analysis

For demographic and clinical characteristics, the Kolmogorov-Smirnov test indicated normally distributed data for the majority of parameters. Two sample t-test and one-way analysis of variance were used to compare continuous variables. In addition, chi-square test was utilized to compare the qualitative variable. All data are expressed as mean ± standard deviation. A p-value less than 0.05 was defined as a significant difference. With regard to nodal properties, 10,000 nonparametric permutation tests were carried out to assess the differences between global and regional network indicators, with age, gender, and education as the covariates^29^. In MDDSI patients, the same method to compare differences in regional topological properties of patients with different levels of suicidal ideation also be used. Moreover, in terms of nodes, Bonferroni correction was performed at the significance threshold of *p* < 0.0005 (0.05/90). In terms of edges, the differences in connection strength were compared between the subnetworks of both groups through network-based statistical (NBS) method^30^ based on rich-club organization. Typically, NBS was corrected by 10,000 non-parametric permutation tests (*P* < 0.01) to estimate the significance of each component in recognizing the connected subnets. The network overlap of MDDSI and MDDNSI is also made into a common network, and this network is also compared with CN through NBS method. Additionally, the connected edges of the difference were specifically put into the above module for observation.

After determining the different nodal properties of the hub nodes in the rich-club organization, partial correlation analysis was employed to evaluate the associations of these hub node/modular of differential connections indicators with HAMD/HAMA scores among all individuals after controlling covariates of age, gender, education, and GM volumes, so as to assess the nodes related to the severity of depression/anxiety.

### Support vector machine (SVM)

The pairwise classification methods of SVM^31^ were applied to MDDSI, MDDNSI, and CN by using the MATLAB-based built-in LibSVM toolbox (http://www.csie.ntu.edu.tw/~cjlin/libsvm). First, each nodal property value and differential connection matrixes are employed as the input features to the classification model. The input data is non-linearly mapped to the high-dimensional feature space. Then a linear separation hyperplane was created in this space to separate two sets of data (MDDSI and MDDNSI, MDDSI and CN, MDDNSI and CN), and the principle of “minimal risk” was adopted to find the best separation hyperplane^32^. Second, the radial basis function (RBF) was selected as the kernel function, and the t-test filter (TF) function was used to filter the difference nodal properties and connections. Third, all the features in the “min-max” scaling method were adopted to ensure that each subject ranged from 0 to 1. Forth, The performance was later compared and visualized according to different metrics including accuracy, specificity, sensitivity, receiver-operating characteristic (ROC) curve, and area under curve (AUC)^33^ via leave-one-out cross-examination (LOOCV). Finally, a permutation test was applied to determine whether the obtained accuracy rate was significant.

## Results

### Demographic data and neuropsychological measures

Table 1 lists the demographic and clinical characteristics of all participants. Differences in age and education among the three groups were not significant (all *P* > 0.05). Additionally, chi-square test demonstrated no significant difference in the ratio of male to female subjects among three groups (*P* > 0.05). As expected, there were significant differences in HAMD among the three groups detected upon ANOVA, meanwhile, post-hoc analysis indicated no significant differences between MDDSI and MDDNSI (*P* > 0.05). For clinical characteristics (disease duration, frequency, and age of onset), two-sample t-test indicated significant differences between MDDSI and MDDNSI (all *P* < 0.05).

**Table. 1:** Demographic and clinical characteristics across all subjects.

	MDDSI (*N* = 69)	MDDNSI (*N* = 58)	CN (*N* = 50)	*P*
Age (years)	40.83 ± 10.89	39.45 ± 11.25	42.48 ± 11.15	0.39
Gender (M/F)	30/39	30/28	24/26	0.34*
Education(years)	10.33 ± 3.82	9.88 ± 3.02	11.24 ± 4.07	0.15
HAMD	30.43 ± 7.37	27.69 ± 6.79	1.36 ± 2.29	<0.001^a,b^
HAMA	16.80 ± 5.30	16.69 ± 7.62	1.43 ± 2.07	<0.001^a,b^
Disease duration (months)	20.55 ± 6.92	18.51 ± 1.87	—	0.12^^^
Disease Frequency (numbers)	3.66 ± 3.11	3.12 ± 2.19	—	0.27^^^
Age of onset (years)	33.43 ± 11.67	32.43 ± 13.15	—	0.65^^^

Note: ^*^P values were obtained using the chi-square test; ^^^*P* values were obtained using two-sample t test, other *P* values were obtained by one-way ANOVA; Unless indicated, data are presented as mean ± standard deviation. Post-hoc analyses were used with least significance difference (LSD) correction (*P* < 0.05): ^a^statistical difference was detected between MDDSI group and CN group; ^b^statistical difference was detected between MDDNSI group and CN group. MDDSI: major depression disorder with suicidal ideation; MDDNSI: major depression disorder with non-suicidal ideation; CN, cognitively normal; M/F: Male/Female; HAMD-17, Hamilton Depression Scale-17 items; HAMA, Hamilton Anxiety Scale.

### Graph theoretical analysis

The properties of SW, network efficiency, and modularity (Mod) at different thresholds of the three groups and the integrated values are shown in Fig. S1 and Tab. S3, respectively. σ > 1 for all groups confirmed the presence of SW organizations with WM connected networks. Moreover, ANOVA revealed significant differences in SW, global efficiency (E_g_), and Mod among the three groups (all *P* < 0.05). Compared with CN, MDDSI patients showed decreased SW (*P* < 0.001), E_g_ (*P* = 0.033) and Mod (*P* = 0.042), whereas MDDNSI patients had reduced SW (*P* < 0.001). In addition, there was no difference between MDDSI and MDDNSI (all *P* > 0.05).

### Network hubs and nodal properties

The hub nodes of rich-club organization with structure network are illustrated in Fig. 1. As observed from the figure, bilateral putamen (PUT), precuneus (PCUN), thalamus (THA), insula (INS), caudate nucleus (CAU), together with left anterior cingulate and paracingulate gyri (ACG.L) were the common hubs in all individuals.

**Fig. 2:**
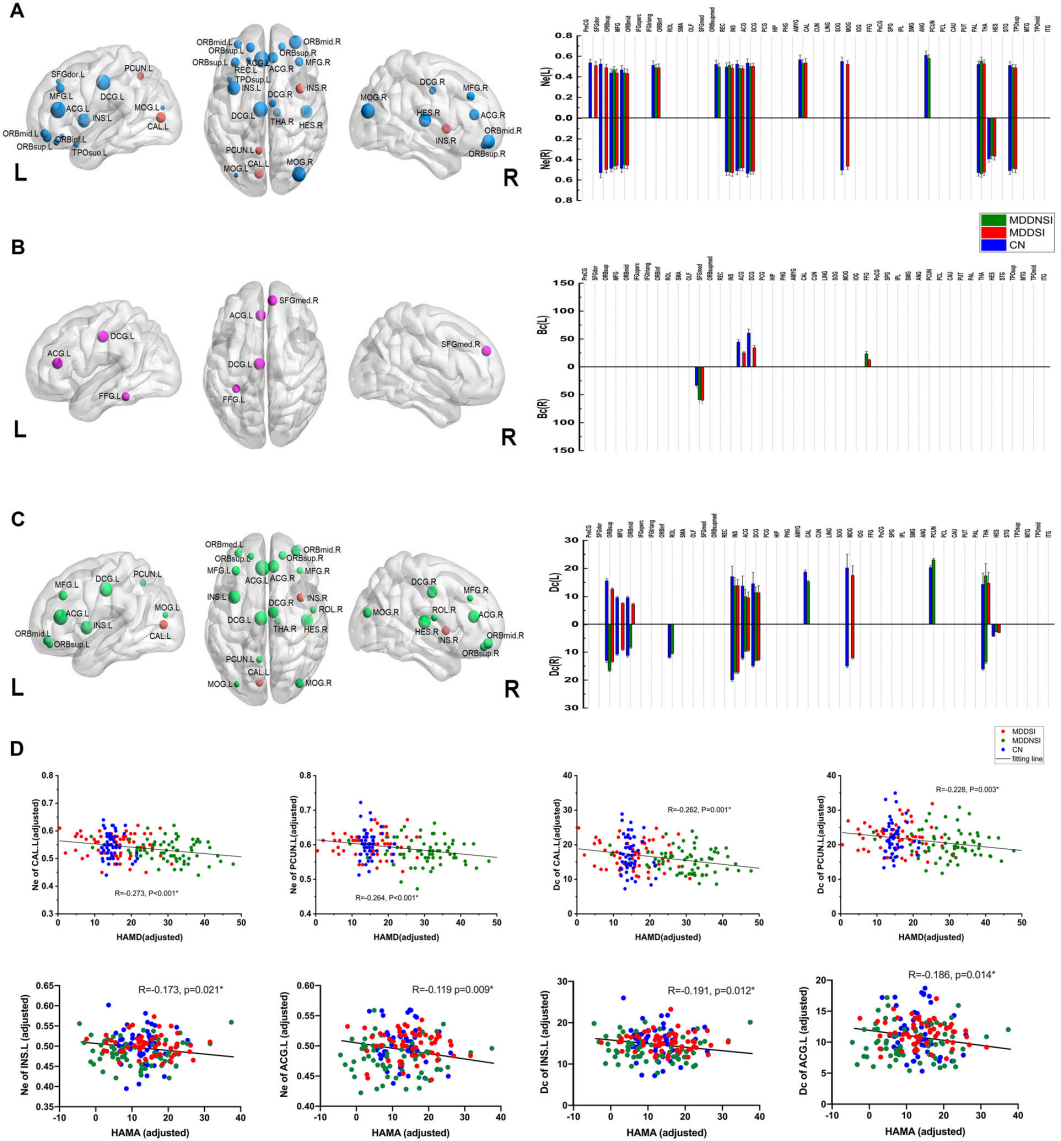
Group-level comparison of nodal properties and behavioral significance. The distribution of the three groups of node property differences (front, **A**: Ne property; **B**: Bc property; **C**: Dc property) and the corresponding nodal values of the left and right hemispheres after the post-hoc analyses (back). The size of the node represents the F value obtained by ANOVA analysis among the three groups. The red node indicates the hub node, and the red asterisk (see **B**) indicates a significant difference between MDDSI and MDDNSI (*P* = 0.0004). These nodes are corrected by Bonferroni correction with a significant different *P* < 0.0005 (0.05/90). The strength of all types of properties is calculated as 20%. **D** The correlation diagram shows the relationship between the different hub node properties (Ne, Bc, Dc) and the severity of depression. The fitted value represents the adjusted value after removing the covariates of age, gender, group, and years of education. CN, cognitively normal; MDDSI, major depression disorder with suicidal ideation; MDDNSI, major depression disorder with non-suicidal ideation. Ne, nodal efficiency; Bc, betweeness centrality; Dc, degree centrality. L. left; R, right; ACG.L, left anterior cingulate and paracingulate gyrus; INS.L, left insula.

Around these common hubs, the nodal properties of brain regions were compared among the three groups (Fig. 2 and Tab. S4). As a result, there were significant differences in Ne and degree centrality (Dc) within the ACG.L, PCUN.L, bilateral THA and bilateral INS regions among the three groups. Additionally, the Bc of ACG.L also showed significant difference. These hubs were mainly involved in the attention circuit (AC) and salience circuit (SC). Post-hoc analyses and hemispherical asymmetry of these nodal properties are exhibited in Fig. 2 and Tab. S5, respectively. Noteworthy, MDDSI patients showed that the Bc property of the left fusiform gyrus (FFG.L) was reduced compared with MDDNSI patients. Besides, Ne, Bc, and Dc manifested left hemisphere asymmetry in these hubs, whereas Ne and Bc also showed left hemisphere asymmetry but Dc asymmetry was not obvious in the non-hubs. Further, in post-hoc analysis, we found that the differential hub regions of the Ne and Dc properties of overlapping MDDSI and MDDNSI were located at bilateral INS, left ACG (ACG.L) and thalamus (THA.L). Regarding the severity of suicide, the differential regional topological nodes are mainly located in non-hubs, and with the exception of olfactory cortex (OLF.L) (*r* = −0.269, *p* = 0.029), the properties of other nodes increase as the severity increases (Fig. S2).

In the core network, the associations between the severity of depression/anxiety and the hub nodal topological properties in all individuals were further assessed (Fig. 2D). It was discovered that the HAMD score was significantly negatively correlated with the hub nodal properties of Ne and Dc (Ne of INS.L, *r* = −0.222, *P* = 0.003; Ne of PCUN.L, *r* = −0.264, *P* < 0.001; Dc of INS.L, *r* = −0.211, *P* = 0.005; Dc of PCUN.L, r = −0.228, P = 0.003). The HAMA score was significantly negatively correlated with the hub nodal properties of Ne and Dc (Ne of INS.L, *r* = −0.173, *P* = 0.021; Ne of ACG.L, *r* = −0.119, *P* = 0.009; Dc of INS.L, *r* = −0.191, *P* = 0.012; Dc of ACG.L, *r* = −0.186, *P* = 0.014). However, there was no significant correlation between Bc and HAMD/HAMA score among all individuals.

### Subnetwork differential connectivity and module analysis

**Fig. 3:**
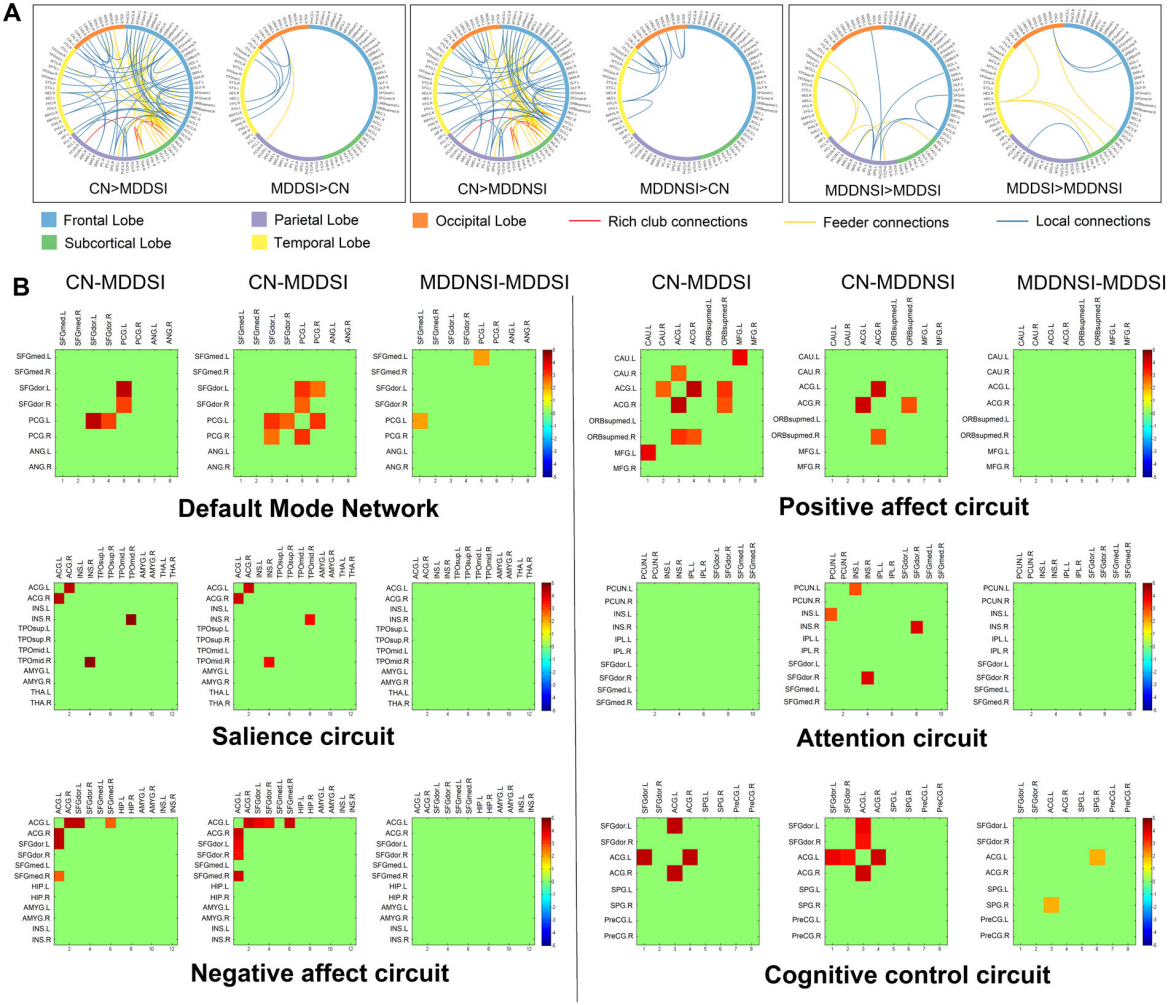
Differential edges of structural subnetworks and the distribution of six related-depression network modules. **A** Differential edges of structural network using the network-based statistic (NBS) method (edge *P* = 0.01, component *P* = 0.1, iteration = 1000) among the three groups. The brain regions are indicated as a circle. The rich club connections (red lines), the feeder connections (yellow lines), and local connections (blue lines) are connected by lines. **B** The differential connection strengths are put into six related-depression network modules. Each small block represents a differential edge of a region connected another region, and the color of the block represents the T value of the corresponding the differential edge. CN, cognitively normal; MDDSI, major depression disorder with suicidal ideation; MDDNSI, major depression disorder with non-suicidal ideation.

Using the two-sample t-test, subnetwork differential connectivity was revealed in the WM connectome (Fig. 3A) related to the six neural circuits (Fig. 3B and Tab. S6). As a result, there was no difference in the rich-club connections between MDDSI and MDDNSI, while the differential connections in local and feeder were related to superior longitudinal fasciculus (SLF) and corpus callosum (CC). Typically, these WM connections were mainly concentrated in the temporal lobe. Compared with CN, the decreased differential brain regions overlap on MDDNSI and MDDSI (Fig. S3) with feeders were mainly located in the frontal and subcortical lobes, while locals were located in the subcortical lobe. Further, the increased differential brain regions were mainly related to feeder connections, which were primarily concentrated in the temporal and occipital lobes. After putting these differential connections into the six neural modules, we found that the difference of WM connectome between MDDNSI and MDDSI was related to the default mode network (DMN) and cognitive control circuit (CCC). In addition, shared MDDNSI and MDDSI differential connections are widely distributed in each module besides the attention circuit (AC). The only differential connection between MDDNSI patients and CN is related to the salience circuit (Fig. S4).

### Classification of SVM

**Fig. 4:**
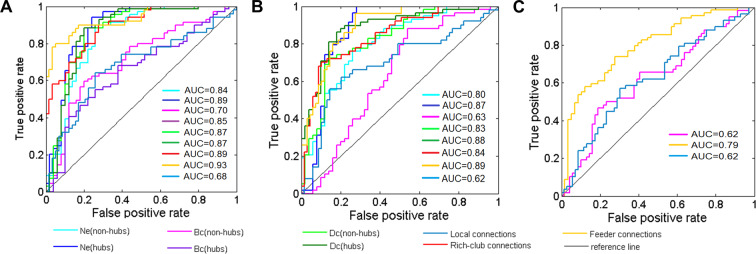
ROC curves from the support vector machine (SVM) method using all topological properties to classify three groups. Nodal properties (non-hubs of Ne, Dc, Bc and their corresponding hubs), and connections (rich-club, feeder, and local) are selected as features. Combining pairwise comparisons **A** MDDSI vs CN, **B** MDDNSI vs CN, **C** MDDSI vs MDDNSI, ROC curves reveal feeder connections have a better classification effect for all indicators. CN, cognitively normal; MDDSI, major depression disorder with suicidal ideation; MDDNSI, major depression disorder with non-suicidal ideation; AUC, area under curve; ROC, receiver operating characteristic curve; Ne, nodal efficiency; Bc, betweeness centrality; Dc, degree centrality.

In this study, the SVM with Gaussian kernel method was employed to facilitate the classification of MDDSI, MDDNSI, and CN. The values of accuracy, sensitivity, and specificity between groups (MDDSI vs CN; MDDNSI vs CN; MDDSI vs MDDNSI) are listed in Tab. S7. As shown in Fig. 4, both nodal properties and connections represent a good power in discriminating MDDNSI and MDDSI patients from CN subjects (AUCs are more than 0.80), except for the Bc and local connections (AUC = 0.63 and 0.62, respectively). It is interesting that the feeder connections offer a better ability to differentiate MDDSI from MDDNSI patients than Bc and local connections (AUC = 0.79 versus 0.62, 0.62). These findings indicated that the feeder represents stronger performance in distinguishing MDDSI from MDDNSI or CN.

## Discussion

In fact, to the best of our knowledge, this is the first study to record the topological convergence and divergence patterns of the core brain WM network in MDD patients with or without SI. As indicated by our observations, firstly, compared to CN subjects, MDDSI and MDDNSI both exhibited disrupted rich-club organizations. However, between MDDSI and MDDNSI, the differential network is concentrated on the non-core low-level network. Secondly, MDDNSI and MDDSI exited similar node and WM fiber deficits pattern. Thirdly, compared with MDDNSI, MDDSI patients showed that the contribution of regional non-hub were significantly destroyed, especially in the FFG.L. The differential structural network connections involved the SLF and CC from feeder and local connections, belong to the CCC and DMN. Finally, the feeder may serve as a potentially powerful indicator for distinguishing MDDSI patients from MDDNSI or CN subjects.

Firstly, the differential nodes between MDDSI and MDDNSI are mainly located in the FFG.L. SI produced a significant effect on the regional properties instead of the global properties of structural network in MDD patients. On the one hand, limited damage in regional network may not be severe enough to affect global measures^33,34^. It may be related to all sub-components of the system following the constraints imposed by other components^35^. Therefore, regional network may be more vulnerable to pathological and physiological characteristics, and potentially serve as biomarkers of early disease stage in MDDSI patients. On the other hand, SI destroys the white matter microstructure of the global network, abnormal nerve fibers may be reconstructed as a compensation mechanism, and the relatively high reentrant connectivity (“cliquishness”) of the regional network increases. In this way, the higher network efficiency of the brain can be maintained^36,37^. Notably, FFG.L is the only brain region in which SI affects the regional network of MDD patients. In fact, it is often involved in the emotional perception of facial stimuli and the resolution of emotional conflict in conflicting task^38^. SI often stems from the perception of negative emotions, abandonment and social disapproval^39^. The abnormalities in fusiform gyrus will compromise the adaptive response to these negative stresses, and suggest that FFG.L might be key component of network circuits reproducing SI.

In addition, the differential connections between MDDSI and MDDNSI are mainly concentrated in SLF and CC, which are located in the DMN and CCC. SLF and CC have been demonstrated in previous studies to be associated with suicide^40,41^, which may help to identify the specific WM fibers associated with MDDSI patients. SI is related to poor decision-making and maladaptive information integration^8,42^. The destruction of DMN leads to a disorder in the processing of comprehensive information related to autobiographical activities (such as autobiography, self-monitoring, and social function), which results in mood disorders and suicidal tendencies. In addition, healthy individuals could bring cognitive flexibility and appropriate social judgment with behavior control^43^. If the CCC is damaged, it will easily cause cognitive rigidity, which deminish their ability to change and adjust their thinking, or course of action. This is critical because it limits the capacity of individuals with suicidal thinking, to produce alternative solutions once they are in predicament, that is, poor decision-making. It is an important feature or precursor of suicide^44^. As discovered from these findings, the structural abnormalities of SLF and CC induced disorders in the DMN and CCC, and mediated the transition from MDDNSI to MDDSI.

As for rich-club organization, it is the central backbone of global communication formed by higher degree nodes and higher connection strengths of inter-nodes^12,45^. In our findings, differential nodes are located on non-hubs, and connections on feeder and local. These are all non-core low-level architectures. The connections of feeder and local in the rich-club organization have been changed in MDD patients with or without SI. Most of these differences are short-range fibers composed of rich-club organization^13,46^. Generally, short-range fibers can provide shortcuts for the connection between adjacent nodes^47^. Although there is no significant difference between feeder and local in MDDSI and MDDNSI patients, the tracked fibers are mainly located in the peripheral regions (feeder and local connections). We speculate that this structural difference in MDD with or without SI is more like a selective vulnerability of regions and connections, rather than overall or global changes. It is more susceptible due to the reduced persistence of peripheral regions and the lower levels in the hierarchical network^48^. However, whether the destruction of node and connections is temporary or continuous, and will be affected by drug treatment, still need the support of future longitudinal studies.

Secondly, compared with CNs, shared nodes and WM fibers alterations in MDDSI and MDDNSI are described thoroughly. For global properties, the decrease in SW reveals a disrupted global integration segregation^49–51^. The combination of divergent hubs in the core network of the three groups is mainly located at PCUN.L, ACG.L, bilateral THA and bilateral INS. Typically, these hubs are related to the integration, processing, and adjustment of emotional information^52–56^. For regional properties, as discovered in post-hoc analysis of the Ne and Dc properties, common hubs of MDDSI and MDDNSI mostly resided in the bilateral INS, ACG.L, and THA.L, which reflected the lower capability and efficiency in the parallel information processes in brain node connectomes^10,57^. Besides, almost all of these nodes are part of the limbic cortico-basal-ganglia circuit^58^. As such, we speculate that a barrier in the communication between the cortico-limbic regions and the subcortical regions may impede the top-down control, leading to unregulated activity in the lower region and causing emotional disturbance^59^. Furthermore, the Ne and Dc properties of ACG.L and INS.L were significantly correlated with severity of depression, indicating that these nodes play a crucial role in the disease development and prognosis of MDD patients with or without SI in the clinical. Furthermore, the results demonstrated that in the overlapping decreased connections of MDDSI and MDDNSI using the NBS method compared with CNs, the feeder was mainly located in the frontal and the subcortical lobes, and the local was located in the subcortical lobe. The reduced connections are more associated with the DMN. Previous studies have shown that functional connections within DMN have increased, and connections between DMN and non-DMN networks also have increased^60^. Regarding the connection between structural networks, it only involved feeder which located in the temporal and occipital lobes. The resting state hypothesis of depression (RSHD)^61^ also provides some speculations for us to study the corresponding symptoms of the structural network modules. For affective symptoms, our study found that, compared with CN, the connections of the bilateral anterior cingulate gyrus (ACG), left ACG and left lateral superior frontal gyrus (SFGdor) related to the positive and negative affect circuits in MDDSI and MDDNSI patients are reduced and overlapped. After the emotional structure network of MDD patients is damaged, it could cause negative information processing bias and concurrent anhedonia. For cognitive symptoms, compared with CN, the connection between left ACG and left SFGdor in MDDSI and MDDNSI patients is reduced. It indicates that the reduction of structural connections in these regions may not promote the internal generation of “mental health” of cognitive activities to foresee many normal events that would occur. For self-focusing symptoms, our research shows that the connection between the left posterior cingulate gyrus (PCG) and the bilateral SFGdor in MDDSI and MDDNSI is reduced compared with CN. It speculates that the destruction of the region might lead to a weakened effect of external stimulation, and internally generated negative stimuli related to resting state activities may become significant. As a result, MDD patients may be locked in a narrow psychological time window^62^, which further enhances people’s sense of despair and ability to focus on themselves. Furthermore, our research showed that compared with CNs, the entire core networks of MDDSI and MDDNSI are damaged. However, two subdivided damaged connections related rich-club rarely overlap. Therefore, we considered that two subtypes of MDD patients may own different core network patterns. The hypothesis needs further research to confirm. In addition, shared connections are mainly belonged to feeder. The middle-high level subnets dysfunction seems to be unable to effectively integrate information from remote brain regions^63^, which may lead to increased sensitivity to pathogenic processes.

More remarkably, our study also proved that relative to CN, the AUC values of hubs of Ne and Dc in MDD patients were higher than those of non-hubs, whereas the feeder values were higher than those of rich and local. The feeder among the three groups had superior predictive power, indicating that WM difference might be used as a candidate neuroimaging biomarker for with or without SI diagnoses. On the whole, the linear SVM classifier performs better than the simple model using topological data only.

Some limitations should be noted in this study. Firstly, this is a cross-sectional study. Therefore, it is difficult to verify the causal relationship between SI and white matter changes. Secondly, deterministic tractography is associated with certain shortcomings in estimating the crossing fibers. Thirdly, classifications featured by nodal properties have low accuracy, which may be related to the availability of too few nodes. Therefore, further efforts should be made to explore the methods involving larger sample subjects, longitudinal design, probabilistic fiber tracking algorithm, and feature combination, so as to overcome the above-mentioned existing problems. Forthly, some patients have received antidepressant drugs that may affect the reported efficacy. Therefore, structural changes caused by pharmacological effects may not be avoided. In order to clarify the specific mechanism of actions, future studies should recruit more medication-naive patients.

In conclusion, using DTI and graph-theoretical analyses, the hierarchical structural network provides clues to the common and unique pathogenesis of MDD patients with or without SI. Moreover, the feeder may be useful as a diagnostic imaging biomarker.

## Supplementary information

supplemental material
