# Supplementary material for: Alterations of core structural network connectome associated with suicidal ideation in major depressive disorder patients
Source: Transl Psychiatry. 2021 Apr 24;11:243. doi: 10.1038/s41398-021-01353-3 (PMC8068724; doi:10.1038/s41398-021-01353-3)
Supplement: Supplementary file 1 — supplemental material [file 41398_2021_1353_MOESM1_ESM.docx]

**SUPPLEMENTARY MATERIAL**

**Title: Alterations of core structural network connectome associated with suicidal ideation in major depressive disorder patients**

Xinyi Liu, Cancan He, Dandan Fan, Feifei Zang, Yao Zhu, Haisan Zhang, Zhijun Zhang, Hongxing Zhang, Chunming Xie

This supplementary material include:

Methods

Table S1-S7

Figure S1-S4

**SUPPLEMENTARY METHODS**

1. **Inclusion and Exclusion Criteria**

In this study, MDD patients were enrolled by a psychiatrist using a structured clinical interview in accordance with the Diagnostic and Statistical Manual of Mental Disorders, 4th Edition (DSM-IV)^1^. MDD patients meeting the following criteria were eliminated from this study: (1) other major psychiatric comorbidities or unstable medical illnesses like schizophrenia, generalized anxiety, cerebrovascular disease, and epilepsy; (2) physical therapy within the past six months and/or antipsychotics (such as antischizophrenics, antidepressants and antianxietics) within the last two weeks; (3) a history of alcohol or drug abuse; and (4) inability to undergo magnetic resonance imaging (MRI) examination.

For CN subjects, psychiatrists comprehensively evaluated their psychiatric conditions to confirm that the current or previous CN subjects did not have any psychopathology. Exclusion criteria that applied to the MDD patients were also applicable to the CN subjects.

1. **Classification of suicidal ideation severity**

If the MDDSI patient scores is 1 in the 3 items of the 17-item Hamilton Depression Rating Scale (HAMD-17) [‘Feels life was not living’], then it is classified as mild MDDSI patients; if the patient scores is 2 [‘wishes he was dead’], then it is classified as moderate MDDSI patients; if the patient scores is 3 [‘suicide ideas’], then it is classified as severe MDDSI patients. Finally, 32 patients with mild MDDSI, 13 with moderate MDDSI and 7 with severe MDDSI, and 17 patients with MDDSI who had no suicidal ideations at this admission were classified.

1. **MRI aquisition and processing**

All MRI data were obtained by the same Siemens Verio 3.0 T scanner (Siemens, Erlangen, Germany) equipped with a standard 12-channel head coil. The DTI sequences involved the following parameters, including repetition time (TR)/echo time (TE) of 10000/90 ms, flip angle (FA) of 90°, field of view (FOV) of 256 × 256 mm^2^, matrix of 128 × 128, 30 diffusion-weighed directions with b of 1000 s/mm^2^, one additional non-dispersion-weighted scan with b of 0, slice thickness of 2 mm, intersection gap of 0, and slice number (interleaved axial) of 70. DTI data were pre-processed with the PANDA package^2^. The DTI image processing procedures were as follows. First of all, brain mask extraction and non-brain space cropping were made. Then, the weighted images were registered on the b0 image for eddy-current distortion and head movement correction through the affine transformation^3^. At last, fractional anisotropy was determined by fitting tensor within each voxel of the diffusion profile^4^. Afterwards, the WM pathways were reconstructed using the deterministic fiber tracking based on fiber assignment.

1. **The specific positioning of the six neural circuit**

Of them, the DMN was defined by combining the anterior medial prefrontal cortex, posterior cingulate cortex, with the angular gyrus^5^. Besides, SC was defined by the core nodes in the anterior cingulate cortex (ACC), anterior insula, and sublenticular extended amygdala^6^. The NAC engaged by the negatively valence stimuli was comprised of subcortical nodes in the amygdala, hippocampus, insula, together with both dorsal and ventral prefrontal nodes^7^. At the same time, the PAC engaged by reward-processing was constituted by the striatal nucleus accumbens ventral tegmental areas, along with their projections to the orbitofrontal cortex and the medial prefrontal cortex^8^. Meanwhile, AC was combined by the nodes in the medial superior frontal cortices, anterior insula, anterior inferior parietal lobule, and precuneus^9^. The CCC was made up of nodes in the dorsolateral prefrontal cortex, ACC, dorsal parietal cortex, and precentral gyrus^10^.

**Reference**

1 First, M. B. Diagnostic and Statistical Manual of Mental Disorders – 4th Ed. (DSM-IV-TR™, 2000). (2000).

2 Cui, Z. *et al.* PANDA: a pipeline toolbox for analyzing brain diffusion images. *Front Hum Neurosci* **7**, 42, doi:10.3389/fnhum.2013.00042 (2013).

3 Irfanoglu, M. O. *et al.* Effects of image distortions originating from susceptibility variations and concomitant fields on diffusion MRI tractography results. *Neuroimage* **61**, 275-288, doi:10.1016/j.neuroimage.2012.02.054 (2012).

4 Chang, L. C., Jones, D. K. & Pierpaoli, C. RESTORE: robust estimation of tensors by outlier rejection. *Magn Reson Med* **53**, 1088-1095, doi:10.1002/mrm.20426 (2005).

5 Greicius, M. D., Krasnow, B., Reiss, A. L. & Menon, V. Functional connectivity in the resting brain: a network analysis of the default mode hypothesis. *Proc Natl Acad Sci U S A* **100**, 253-258, doi:10.1073/pnas.0135058100 (2003).

6 Seeley, W. W. *et al.* Dissociable intrinsic connectivity networks for salience processing and executive control. *J Neurosci* **27**, 2349-2356, doi:10.1523/JNEUROSCI.5587-06.2007 (2007).

7 Kober, H. *et al.* Functional grouping and cortical-subcortical interactions in emotion: a meta-analysis of neuroimaging studies. *Neuroimage* **42**, 998-1031, doi:10.1016/j.neuroimage.2008.03.059 (2008).

8 Haber, S. N. & Knutson, B. The reward circuit: linking primate anatomy and human imaging. *Neuropsychopharmacology* **35**, 4-26, doi:10.1038/npp.2009.129 (2010).

9 Fornito, A., Harrison, B. J., Zalesky, A. & Simons, J. S. Competitive and cooperative dynamics of large-scale brain functional networks supporting recollection. *Proc Natl Acad Sci U S A* **109**, 12788-12793, doi:10.1073/pnas.1204185109 (2012).

10 Cole, M. W. & Schneider, W. The cognitive control network: Integrated cortical regions with dissociable functions. *Neuroimage* **37**, 343-360, doi:10.1016/j.neuroimage.2007.03.071 (2007).

**FIGURE LEGENDS**

**Fig.S1 Group-level comparison of global and modular topological properties in the structural network among MDDSI, MDDNSI and CN subjects.** **(A)** Group-level comparison of global and modular measures under different sparsity. The range of sparse thresholds is 0.05-0.2. Asterisks indicate statistical difference among 3 groups using ANOVA analysis (*P* < 0.05) (Bonferroni correction). **(B)** Group-level comparison of functional integration measures in the structural network. The error bars indicate standard deviation. ^*^, post-hoc analyses were corrected by Bonferroni correction with a significant different *P*<0.017 (0.05/3). **Abbreviation:** CN, cognitively normal; MDDSI, major depression disorder with suicidal ideation; MDDNSI, major depression disorder with non-suicidal ideation; Cp, clustering coefficient; L, shortest path length; SW, small world; E_loc_, local efficiency; E_g_, global efficiency.

**Fig.S2 Association between the severity of suicidal ideation in MDDSI patients as well as topological properties of whole brain nodes.** Abbreviation: IFGtriang, Inferior frontal gyrus, triangular part; AMYG, Amygdala; MOG, Middle occipital gyrus; OLF, Olfactory cortex; L, left; R, right; MDDSI, major depression disorder with suicidal ideation.

**Fig.S3 Shared MDDSI and MDDNSI differential edges of structural subnetworks.** Compared with CNs, the decreased network of shared MDDSI and MDDNSI is shown in **Fig.A**, and the increased network is shown in **Fig.B**. The shared MDDSI and MDDNSI differential edges of structural network using the network-based statistic (NBS) method (edge P = 0.01, component P = 0.1, iteration = 1000) compared with CN. The brain regions are indicated as a circle. The feeder connections (yellow lines) and local connections (blue lines) are connected by lines.

**Fig.S4 Association between the module of the differential white matter fiber connection strength as well as severity of depression/anxiety.** Colorbar stands for correlation significance. FA, fractional anisotropy; HAMD, Hamilton Depression Scale.
